# Pharmacological Modulation of β-Catenin Preserves Endothelial Barrier Integrity and Mitigates Retinal Vascular Permeability and Inflammation

**DOI:** 10.3390/jcm12227145

**Published:** 2023-11-17

**Authors:** Madhuri Rudraraju, Shengshuai Shan, Fang Liu, Jennifer Tyler, Ruth B. Caldwell, Payaningal R. Somanath, S. Priya Narayanan

**Affiliations:** 1 Clinical and Experimental Therapeutics, Clinical and Administrative Pharmacy Department, College of Pharmacy, University of Georgia, Augusta, GA 30912, USA; 2 Research and Development, Charlie Norwood VA Medical Center, Augusta, GA 30912, USA; 3 Vision Discovery Institute, Augusta University, Augusta, GA 30912, USA; 4 Vascular Biology Center, Augusta University, Augusta, GA 30912, USA

**Keywords:** retinal inflammation, vascular permeability, blood-retinal barrier, β-catenin, lipopolysaccharide, ICG001

## Abstract

Compromised blood-retinal barrier (BRB) integrity is a significant factor in ocular diseases like uveitis and retinopathies, leading to pathological vascular permeability and retinal edema. Adherens and tight junction (AJ and TJ) dysregulation due to retinal inflammation plays a pivotal role in BRB disruption. We investigated the potential of ICG001, which inhibits β-catenin-mediated transcription, in stabilizing cell junctions and preventing BRB leakage. In vitro studies using human retinal endothelial cells (HRECs) showed that ICG001 treatment improved β-Catenin distribution within AJs post lipopolysaccharide (LPS) treatment and enhanced monolayer barrier resistance. The in vivo experiments involved a mouse model of LPS-induced ocular inflammation. LPS treatment resulted in increased albumin leakage from retinal vessels, elevated vascular endothelial growth factor (VEGF) and Plasmalemmal Vesicle-Associated Protein (PLVAP) expression, as well as microglia and macroglia activation. ICG001 treatment (i.p.) effectively mitigated albumin leakage, reduced VEGF and PLVAP expression, and reduced the number of activated microglia/macrophages. Furthermore, ICG001 treatment suppressed the surge in inflammatory cytokine synthesis induced by LPS. These findings highlight the potential of interventions targeting β-Catenin to enhance cell junction stability and improve compromised barrier integrity in various ocular inflammatory diseases, offering hope for better management and treatment options.

## 1. Introduction

Ocular injuries, responsible for a significant proportion of ophthalmic emergencies [1], arise from inflammation and vascular permeability as the underlying causes of diabetic retinopathy (DR), age-related macular degeneration, macular edema associated with diabetes, uveitis, and retinal vein occlusion [2,3,4]. Although the pathological permeability in the eye is mostly vascular endothelial growth factor (VEGF)-driven [5] and anti-VEGF therapy remains the gold standard for treating retinal edema, some patients show a sub-optimal response to anti-VEGF therapy, highlighting underlying chronic VEGF-independent inflammation [6]. Corticosteroids and other anti-inflammatory agents are often beneficial as adjunctive treatments for retinal edema, suggesting vascular permeability and inflammation are closely related and, at times, bi-directional [7]. Studies report that a proportion of cytokines and chemokines mediate ocular inflammation and contribute to proliferative diabetic retinopathy in a VEGF-independent manner [8]. The molecular mechanisms downstream of VEGF that lead to barrier disruption are an active area of investigation [9]. While it is known that the loss of cell junctions contributes to the development of pathological vascular permeability in several disease states, the changes observed in these proteins associated with vascular endothelial hyperpermeability and inflammation are still unclear.

The loss of junctional integrity and the subsequent breakdown of the BRB are considered some of the key initiating phases of vascular permeability and retinal edema [10]. The loss of barrier function contributes to the exacerbation of inflammation [11]. Several studies have reported that vascular hyperpermeability due to the loss of paracellular cell junctional integrity or increased transcytosis is often associated with inflammatory cell activation in the perivascular area [12,13,14]. Nevertheless, there remains a void in understanding cell junction protein alterations in retinal inflammation and how they influence BRB integrity. Pharmacological means to restore BRB could serve as a potential therapeutic modality to suppress retinal vascular permeability and inflammation.

A key component of endothelial adherens junction (AJs) is VE-cadherin, which mediates homophilic interactions between endothelial cells (ECs) [15] and acts as a communicating bridge with VEGF signaling [16,17]. VE-cadherin triggers the activation of intracellular molecules, induces Rac activation [18], promotes the phosphoinositide 3-kinase (PI3K)-Akt pathway, and stabilizes cell–cell contacts [19] by promoting the synthesis of endothelial claudin-5 (Cldn5) [20]. In addition, VE-cadherin is critical in the regulation of permeability in response to VEGF and inflammatory cytokines [15,21,22].

β-catenin is a critical component of AJs but also an essential mediator of Wnt signaling [23,24]. Our previous studies have demonstrated that a significant portion of β-catenin within human retinal endothelial cells (HRECs) is primarily located at the cell–cell junctions. This localization is notably perturbed when HREC barrier integrity is compromised [25]. The literature indicates that a fraction of intracellularly localized β-catenin also participates in the Wnt signaling pathway, leading to the production of pro-inflammatory markers and cytokines [26]. The role of β-catenin in retinal ECs is not fully characterized. Identifying ways to improve AJ stability, improve β-catenin membrane localization, and prevent its internalization [27] can help in correcting vascular leakage. Simultaneously, directing interventions towards the inhibition of Wnt-mediated β-catenin nuclear translocation may also prove beneficial in suppressing the release of pro-inflammatory cytokines. While several studies emphasize how AJ destabilization results in pathological vascular permeability [28,29,30,31], there are limited studies on how AJs can be pharmacologically stabilized. Specifically, an option to target β-catenin to stabilize AJs and suppress Wnt-mediated inflammation for therapy is unexplored.

ICG001 selectively targets the β-catenin-mediated transcription by disrupting its interaction with CREB binding protein [32]. Consequently, ICG001 serves as a valuable tool for the precise investigation of the significance of the β-catenin-mediated transcription in the context of Wnt and AJ signaling. Previous studies from our lab showed how ICG001 was beneficial in preventing endothelial-to-mesenchymal transition in vitro and in vascular remodeling in a chronic hypoxia model in mice [33,34]. We posited that treatment with ICG001 will translocate β-catenin from the nucleus to the AJs, which, in turn, is a prerequisite for fostering interactions between the VE-Cadherins of neighboring cells, ultimately resulting in AJ stabilization. Using lipopolysaccharide (LPS)-treated HRECs, and an experimental model of LPS-induced retinal inflammation in mice, we investigated the effect of nuclear β-catenin inhibition on BRB regulation and on suppression of inflammation. Our studies revealed the potential therapeutic benefits of inhibiting β-catenin transcriptional activity in LPS-induced retinal inflammatory injury.

## 2. Materials and Methods

### 2.1. Cell Culture and Reagents

Primary Human retinal ECs (HRECs; Cat No. H1168) were purchased from Cell Biologics (Chicago, IL, USA). Cells were maintained in EC Basal Medium (Cat No. H1168- Cell Biologics, Chicago, IL, USA) fortified with EC growth supplements, antibiotics, and fetal bovine serum. Cells were grown on flasks pre-coated with gelatin (0.2%). Cells from passages 6 to 10 were used for experiments. Lipopolysaccharide (LPS) lyophilized powder (Cat No. L2018) was obtained from Millipore Sigma (Boston, MA, USA). ICG001 (Foscenvivint—Cat No. S2662) was obtained from Selleckchem (Houston, TX, USA).

### 2.2. Measurement of Endothelial-Barrier Resistance

Endothelial resistance as a sign of barrier integrity was measured using the electric cell–substrate impedance sensing (ECIS) equipment (Applied Biophysics, Troy, NY, USA) as described previously [25,35]. Before treating the cells, HREC monolayer basal resistance was measured to allow synchronization and achieve a stable resistance. Once stable resistance was recorded, cells were treated with LPS (0.1 µg/mL) or ICG001 (10 µM) for 24 h, and the endothelial–barrier resistance was measured in real-time at multiple frequency modes.

### 2.3. Animals

We utilized male and female C57BL6J mice, aged 8 to 10 weeks, procured from Envigo (Indianapolis, IN, USA). Our experimental protocols adhered to the guidelines established by the Association for Research in Vision and Ophthalmology (ARVO) for the ethical treatment of animals in ophthalmic and vision research. The Institutional Animal Care and Use Committee of the Charlie Norwood VA Medical Center, Augusta, GA, USA, approved all procedures (protocol #1604232). We made every possible effort to minimize pain and ensure the welfare of the animals throughout the experimental procedures.

### 2.4. LPS-Induced Retinal Inflammation Model, ICG001 Treatment, and Sample Collection

**Figure 1:**
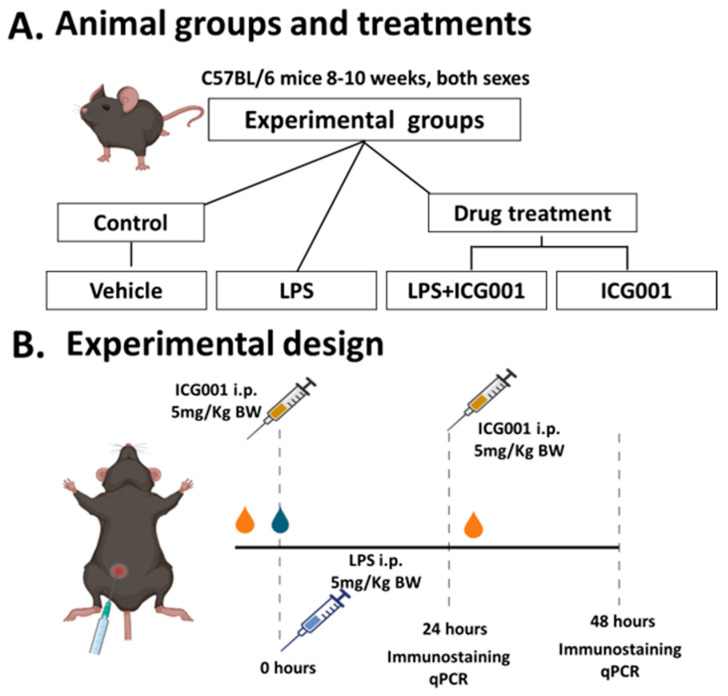
Experimental design of LPS-induced retinal inflammation and ICG001 treatment schedule. (**A**) Adult wild-type (C57BL6J) mice were treated with LPS to induce ocular inflammation. ICG001 or vehicle (PBS + DMSO) was given an hour before LPS injection. (**B**) The effects of the drug were studied at two different time points (24 and 48 h). Immunostaining was performed using albumin, microglia/macrophage (Iba1, F4/80), and macroglia (GFAP) markers. RT-PCR analysis was performed to determine the expression pattern of several inflammatory mediators. LPS: lipopolysaccharide, i.p.: intraperitoneal injection, qPCR: quantitative PCR.

We used an LPS-induced inflammation model to study ocular inflammation in mice with clinical and histopathological similarities to acute anterior uveitis in humans [36,37]. LPS-induced retinal inflammation was achieved by giving a single intraperitoneal (i.p.) injection of LPS at a 5 mg/Kg dose. Control mice received phosphate buffer saline (PBS) as vehicle treatment. Next, we examined the effects of ICG001 to combat vascular permeability and inflammation in this acute setting. The drug was administered one hour before LPS treatment at a dose proven to be effective and non-toxic (5 mg/kg, i.p.) in other animal studies [34]. We investigated the effect of ICG001 at two time points (24 and 48 h). For the 24 h time point study, mice received a single dose of LPS or ICG001. In the 48 h time-point study, mice received a single dose of LPS and two doses of ICG001 at 0 and 24 h. The treatments resulted in eight groups (4 each for 24 and 48 h study) of mice as presented in Figure 1A.

Following trans-cardiac perfusion using PBS, mouse eyeballs were collected and fixed in 4% paraformaldehyde at 4 °C overnight, washed in PBS, and cryopreserved in 30% sucrose (4 °C for 48 h or until they sank to the bottom of the tubes). Eyeballs were carefully embedded in an OCT medium (Cat No. 4583, Tissue-Tek^®^, Tokyo, Japan), snap-frozen, and stored at −80 °C for further histological processing, and retinal cryosections (10 µm thickness) were prepared. A schematic of animal experiments is provided in Figure 1B.

### 2.5. Immunofluorescence Staining

Immunofluorescence staining of HRECs was performed as previously described [25]. Approximately, 1 × 10^4^ cells per mL of media were seeded into 8 well chamber slides (Cat No. 154461PK, Thermo Scientific™, Waltham, MA, USA) and allowed to grow to confluence, following treatment with LPS or ICG001 for 24 h; cell monolayers were washed in PBS. The cells were then fixed by ice-cold 4% PFA (Cat No. AAJ19943K2, Affymetrix, Santa Clara, CA, USA) for 20 min., washed (PBS), and permeabilized in 0.2% Triton X-100 for 15 min. Cells were further incubated in a blocking solution (10% normal donkey serum containing 0.5% Triton-X in PBS) for one hour. They were then incubated in primary antibodies (Table 1) at 4 °C overnight. The next day, the samples were incubated (1 h) with respective secondary antibodies (Table 1). The chamber slides were washed in PBS and cover slipped using mounting medium (Vectashield (Cat. no. H-1000, Vector Laboratories, Newark, CA, USA).

Immunostaining of retinal cryostat sections was carried out according to the methods established in our laboratory [38,39]. Briefly, the sections were permeabilized in Triton X-100 (0.2%, 15 min), blocked in normal goat serum (10% NGS, for one hour), washed (PBS), and incubated in respective primary antibodies (Table 1) at 4 °C overnight. The next day, the sections were washed in PBS followed by incubation (1 h) in respective fluorescein-conjugated secondary antibodies in the absence or presence of GS-IB4 (Isolectin B4 from *Griffonia simplicifolia*) for vascular staining (Table 1). The sections were subsequently washed and mounted using Vectashield (Cat. no. H-1000, Vector Laboratories). Image acquisition was conducted using an LSM 980 ZEN 3.1 confocal microscope (Zeiss, Oberkochen, Germany).

### 2.6. Measurement of Vascular Permeability Using Albumin Immunostaining

Increased vascular permeability in mice after treatment with LPS was studied by measuring the extravasation of albumin [40]. Mice were intracardially perfused with PBS (to clear blood from tissues) and then with 4% paraformaldehyde in PBS for fixing tissues. The magnitude of albumin leakage from retinal blood vessels was determined by immunostaining of retinal sections for albumin and quantifying the intensity of extravasated albumin immunofluorescence which was normalized to the control group.

### 2.7. Quantification of Immunofluorescence

Fluorescence intensity quantification of immunostained retinal sections was performed using NIH ImageJ software (version 1.31) and expressed as the fluorescence intensity per field of view (Fov) for each marker used. The area of fields measured per marker was maintained uniformly throughout all the groups and values were normalized relative to the control group. A minimum of three sections (20 µm apart) per mouse were immunostained.

### 2.8. RNA Isolation and Quantitative RT-PCR

The RNA isolation and analysis of retinal tissues were carried out as previously described [40]. Briefly, the retinal tissue was homogenized using a Micro-Tube homogenizer (Cat No. F65100-0000, SP BEL-ART scientific products) using QIAzol Lysis Reagent (Cat No.79306, Qiagen, Hilden, Germany). Total RNA extraction was performed using miRNeasy mini kit (Cat No. 217084, Qiagen). Around 500 ng of total RNA was used for cDNA synthesis using a High-Capacity cDNA Reverse Transcription Kit (Cat No. 4368814, Applied Biosystems, Waltham, MA, USA). Quantitative PCR was carried out with StepOnePlus™ Real-Time PCR System (Cat No. 4376600, Applied Biosystems) using Power SYBR Green Master Mix (Cat No. 4309155, Applied Biosystems). Data were normalized to GAPDH or HPRT, and the fold change was calculated by the ΔΔCt method. The primers used in the study are presented in (Table 2).

### 2.9. Statistical Analysis

Statistical analysis was performed using GraphPad Prism 9 software. One-way ANOVA was followed by the Tukey test for multiple comparisons. Results with *p* < 0.05 were considered significant.

## 3. Results

### 3.1. Treatment with ICG001 Strengthened the HREC Barrier by Improving β-Catenin Junctional Distribution

**Figure 2:**
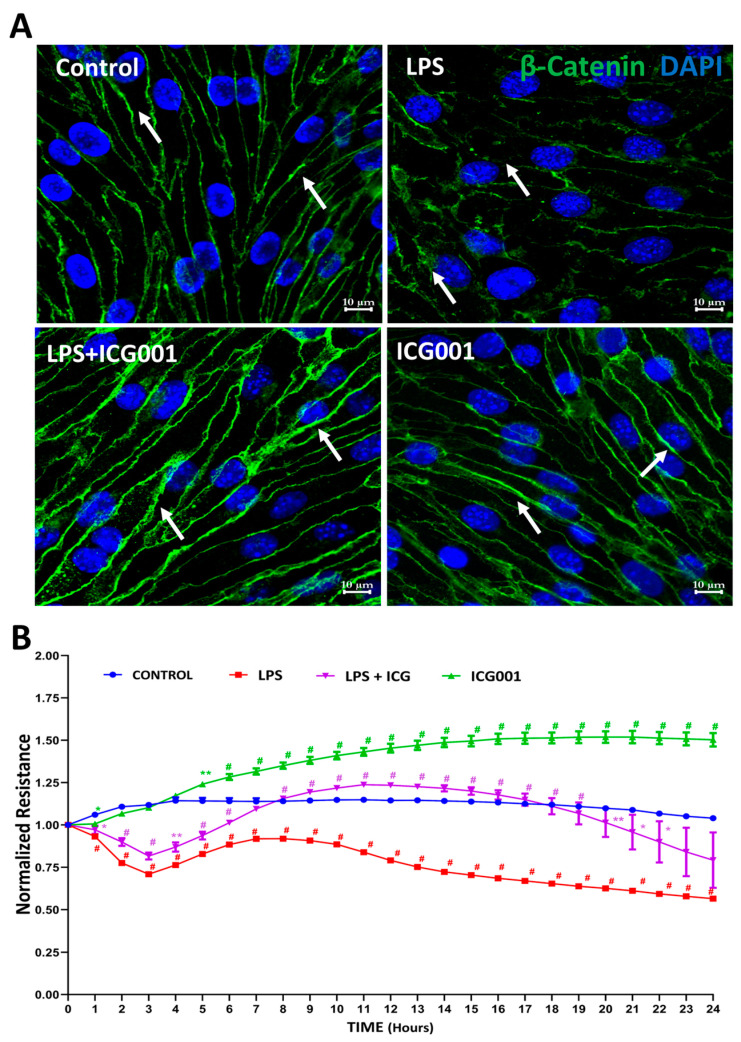
Treatment with ICG001 promoted β-catenin recruitment in AJs to enhance HREC barrier resistance. (**A**) Representative images of immunostained HREC monolayers with anti-β-catenin antibodies showing reduced localization of β-catenin in AJs with LPS treatment and improved recruitment of β-catenin in AJs with ICG001 treatment (*n* = 5). Scale Bar: 10 µm. Arrows indicate the junctional staining of β-catenin. (**B**) Bar graph showing improved HREC barrier resistance with ICG001 treatment in ECIS analysis (*n* = 3–4). Data comparisons were LPS or ICG001 vs. Control and LPS + ICG001 vs. LPS; data shown as mean ± SEM. * *p* < 0.01, ** *p* < 0.001, # *p* < 0.05.

Primary HREC monolayers exposed to LPS (0.1 µg/mL) treatment for 24 h and immunostained for β-catenin revealed substantial disruption of β-catenin at the junctional space (Figure 2A). Concurrent administration of ICG001 (10 µM) reinstated normal β-catenin junctional distribution, a phenomenon also corroborated by the observed increase in barrier resistance of HREC monolayers following ICG001 treatment (Figure 2B). In contrast, treatment with LPS alone significantly diminished the barrier resistance of HREC monolayers (Figure 2B). These findings reveal the potential of β-catenin stabilization using ICG001 to enhance HREC barrier integrity by improving the junctional distribution of β-catenin.

### 3.2. Treatment of Mice with ICG001 Blocked LPS-Induced Retinal Vascular Leakage

**Figure 3:**
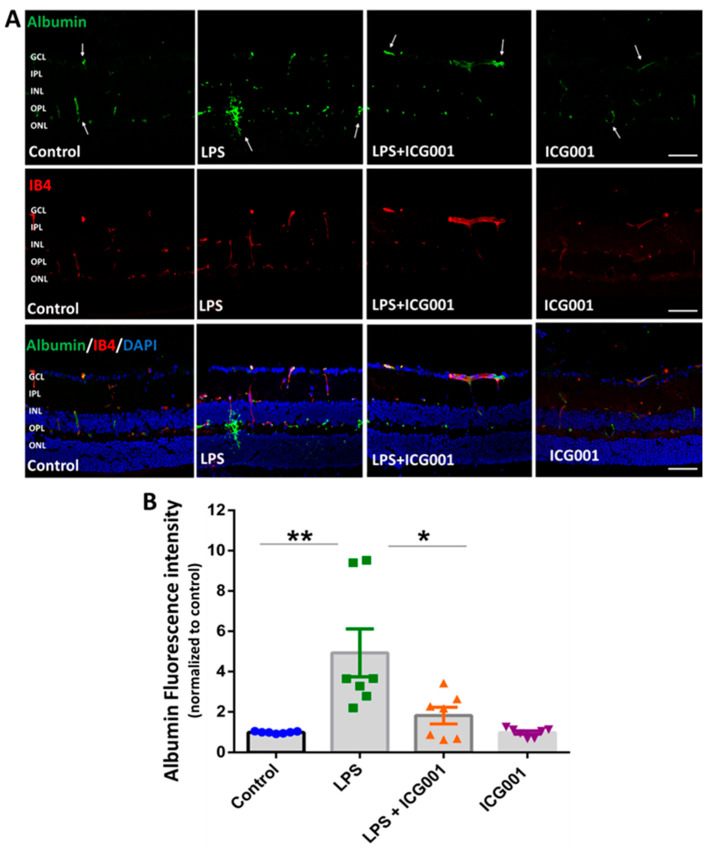
ICG001 treatment reduced LPS-induced albumin extravasation from retinal blood vessels. (**A**) Representative images of retinal sections immunostained with anti-albumin antibody showing vascular leakage with LPS treatment at 24 h. IB4 was used to stain the blood vessels. Co-treatment with ICG001 significantly reduced LPS-induced vascular leakage in the mouse retinas. Arrows indicate areas of albumin extravasation. (**B**) Mean fluorescence intensity of albumin staining quantified per field of view (*n* = 7–8/group, ** *p* < 0.01, * *p* < 0.05; data presented as mean ± SEM); scale bar: 50 µm.

Albumin immunostaining, along with IB4 staining (facilitating the visualization of blood vessels), was used to study vascular leakage in the retina [41]. Mice were treated with LPS, ICG001, or a combination of both for 24 h. Alterations in retinal vascular permeability were assessed with immunolocalization of extravasated albumin, co-stained with IB4, and viewed through confocal microscopy. While LPS treatment elevated albumin extravasation in the deeper retinal capillaries, concurrent administration of ICG001 significantly inhibited this effect. (Figure 3A). The mice treated with ICG001 alone or the vehicle control displayed no albumin extravasation in the retina. Retinal vascular leakage was quantified by measuring the fluorescence intensity of extravasated albumin per field of view (Figure 3B). These combined findings demonstrate the ability of ICG001 to effectively attenuate LPS-induced retinal vascular permeability.

### 3.3. Treatment of Mice with ICG001 Blunted the LPS-Induced Increase in Retinal Pro-Inflammatory Cytokines and Chemokines

**Figure 4:**
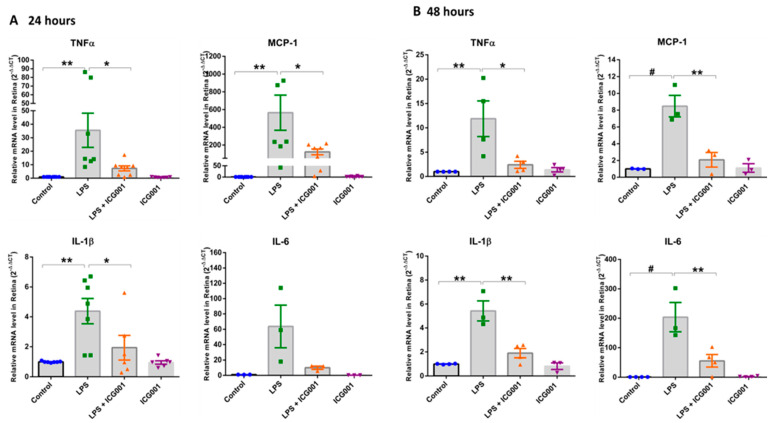
ICG001 treatment blunted LPS-induced pro-inflammatory cytokine expression in the mouse retinas. Quantitative RT-PCR analysis of mice retinas demonstrated a significant increase in the mRNA levels of pro-inflammatory cytokines TNFα, MCP–1, IL–1β, and IL–6 upon treatment with LPS at (**A**) 24 h and (**B**) 48 h, which were attenuated with ICG001 treatment. (*n* = 6–8/group for 24 h and *n* = 3–4/group for 48 h samples), # *p* < 0.001, ** *p*< 0.01, * *p* <0.05; data presented as mean ± SEM).

Retinal q-RT PCR analysis revealed elevated mRNA levels of pro-inflammatory cytokines and chemokines in LPS-treated mice, suggesting a robust inflammatory surge in the retina at both 24 and 48 h (Figure 4). LPS-induced upregulation in TNFα, MCP-1, IL-1β, and IL-6 was significantly inhibited in ICG001 co-treated groups. Treatment with ICG001 alone did not show any changes in the expression of pro-inflammatory cytokines and chemokines. These findings suggest ICG001 could limit the LPS-induced inflammatory surge in the retina.

### 3.4. ICG001 Treatment Ameliorated LPS-Induced Microglia/Macrophage Activation

**Figure 5:**
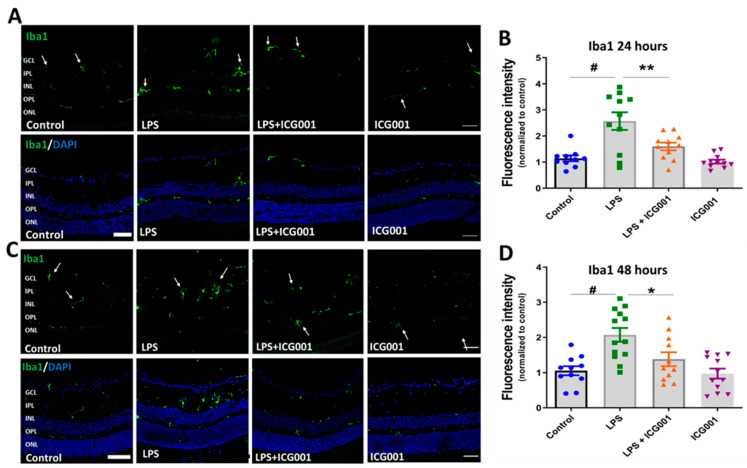
ICG001 treatment inhibited LPS-induced increases in Iba1-positive macrophage/microglia in the retina. Representative images of retinal sections immunostained with anti-Iba1 antibodies at 24 (**A**) and 48 h (**C**) revealed increased Iba1 immunoreactivity with LPS treatment, which was significantly inhibited with ICG001 co-treatment. Arrows indicate Iba1 positive cells. (**B**,**D**) Mean fluorescence intensity quantified per field of view (*n* = 9–12/group), # *p* < 0.001, ** *p* < 0.01, * *p* < 0.05; data presented as mean ± SEM); scale bar: 50 µm.

**Figure 6:**
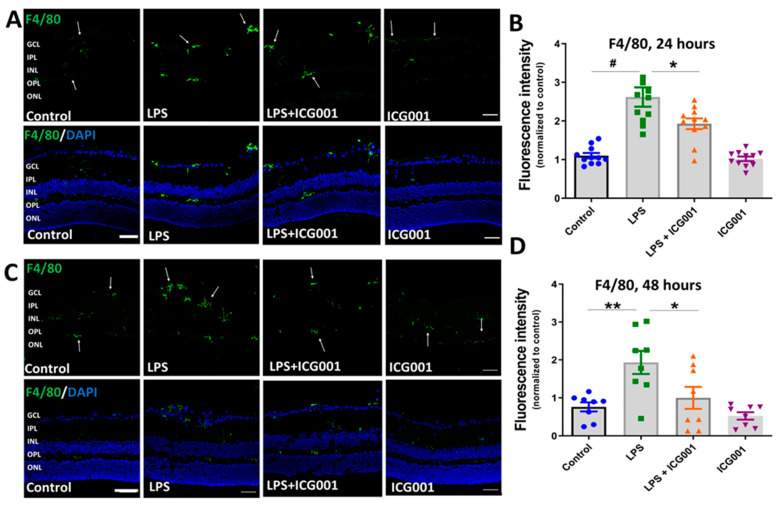
ICG001 treatment inhibited LPS-induced increase in F4/80 positive microglia/macrophages in the retina. (**A**,**C**) Representative confocal images of retinal cryostat sections immunostained with anti-F4/80 antibodies at 24 and 48 h. Results show increases in F4/80 immunoreactivity in response to LPS treatment, which is blunted upon co-treatment with ICG001. Arrows indicate F4/80 positive cells. (**B**,**D**) Mean fluorescence intensity quantified per field of view (*n* = 8–11/group), # *p* < 0.001, ** *p* < 0.01, * *p* < 0.05; data presented as mean ± SEM; scale bar: 50 µm.

We investigated the changes in immunoreactivity of the microglia/macrophage markers Iba1 and F4/80 in the retina in response to treatment with LPS, ICG001, and a combination of both. LPS treatment is reported to induce microglia/macrophage activation in the retina [42]. Immunostaining of the retinal cryostat sections (24 and 48 h) using Iba1 (Figure 5) or F4/80 (Figure 6) showed an increase in the number of cells positive for the microglia/macrophage markers in LPS-treated groups. The cells positive for Iba1 or F4/80 were localized in the inner plexiform layer (IPL), inner nuclear layer (INL), or outer plexiform layer (OPL) of the retina (indicated by arrows). However, co-treatment with LPS and ICG001 markedly reduced these changes in the retina at both 24 and 48 h time points (Figure 5 and Figure 6). ICG001 treatment alone did not alter the status of microglia/macrophage as compared to the vehicle-treated groups. Quantification of fluorescence intensity of Iba1 or F4/80 expression was used as a measure of microglia/macrophage status. LPS treatment significantly upregulated the expression of Iba1 and F4/80 at both 24 and 48 h time points, and the immunoreactive cells are more numerous with the LPS treatment. These effects were significantly reduced by ICG001 treatment (Figure 5 and Figure 6). These results reveal the potential effect of ICG001 in reducing microglia/macrophages positive for Iba1 or F4/80 in the retina.

### 3.5. LPS-Induced Retinal Gliosis Was Attenuated by ICG001 Treatment

**Figure 7:**
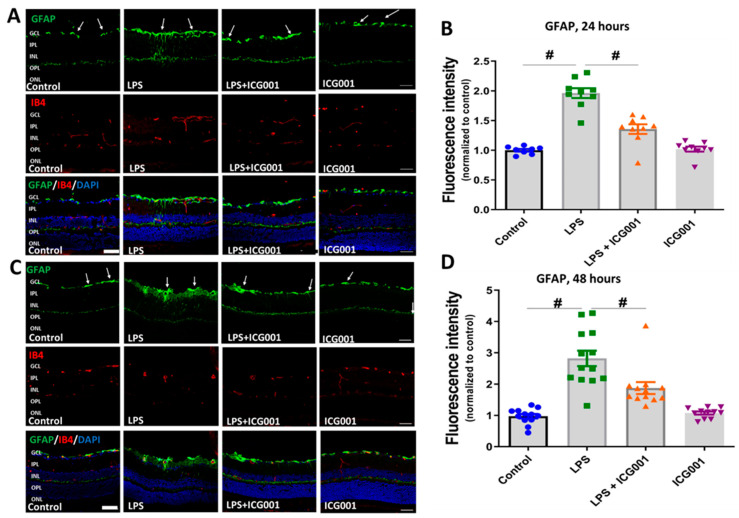
ICG001 treatment ameliorated LPS-induced retinal gliosis. (**A**,**C**) Representative confocal images of GFAP immunostaining of the retinal sections at 24 and 48 h. Upregulation of GFAP expression is evident in macroglia (Müller cells and astrocytes) in response to LPS treatment which is reduced by co-treatment with ICG001. IB4 was used to stain the blood vessels. Arrows indicate representative areas with GFAP immunoreactivity. (**B**,**D**) Mean fluorescence intensity quantified per field of view (*n* = 8–13/group), # *p* < 0.01; data presented as mean ± SEM; scale bar: 50 µm.

Astrocytes and Müller cells comprise the macroglia of the retina. Glial Fibrillary Acidic Protein (GFAP) is widely used as a marker to study retinal gliosis. GFAP expression is elevated in Müller cells in response to retinal stress/injury. Our results show a significant increase in retinal gliosis at 24 and 48 h following LPS treatment, as studied by GFAP immunofluorescence staining. In the control and ICG treated retinas GFAP expression was observed in the GCL area indicating the astrocytic expression. However, in response to LPS treatment, elevated GFAP expression was observed in GCL and in the inner retina, representing astrogliosis as well as activation of Müller cells in the LPS-treated mice. Interestingly, treatment with ICG001 markedly reduced these changes at 24 and 48 h (Figure 7), suggesting that ICG001 can suppress LPS-induced gliosis in the retina.

### 3.6. Treatment with ICG001-Modulated, LPS-Induced VEGF and PLVAP Expression in the Retina

**Figure 8:**
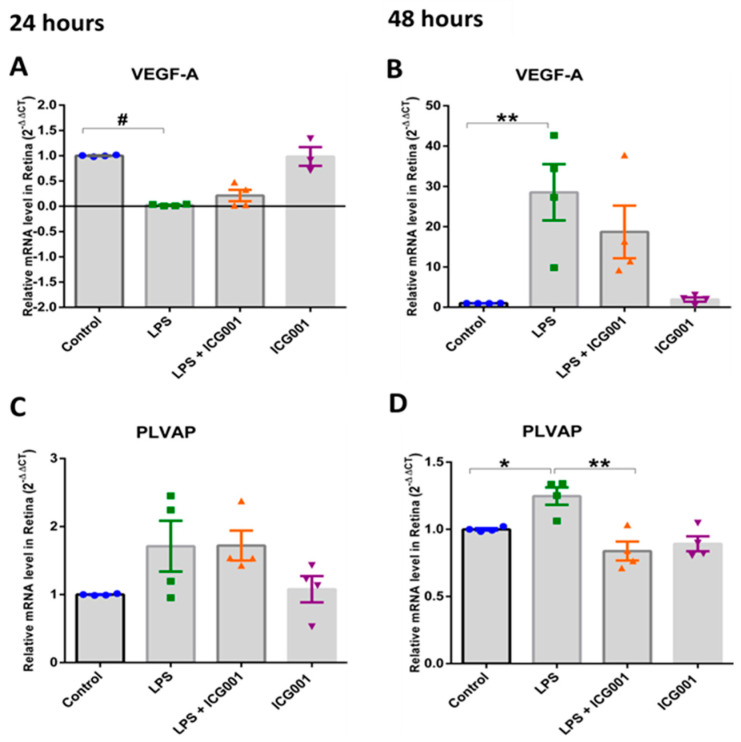
Late-stage elevation in VEGF and PLVAP expression by LPS is inhibited by ICG001 treatment. (**A**,**C**) Quantitative RT-PCR analysis of mouse retinas demonstrated modest variations in VEGF and PLVAP mRNA expression with LPS treatment in the mouse retinas after 24 h. (**B**,**D**) Significant increase in VEGF and PLVAP mRNA expression in LPS-treated mouse retinas was observed after 48 h, which is reversed by co-treatment with ICG001 (*n* = 3–4/group for 24 h, *n* = 3–4/group for 48 h samples), # *p* < 0.001,** *p* < 0.01, * *p* < 0.05; data presented as mean ± SEM).

Next, we assessed alterations in the expression of two vital molecules implicated in the regulation of endothelial barrier function, VEGF and PLVAP, in response to LPS with and without co-treatment with ICG001. While a modest decrease in VEGF mRNA level was observed at the 24 h time point with LPS insult, the mRNA level of VEGF was increased by almost 20-fold at the 48 h time point. Intriguingly, treatment with ICG001 slightly reduced VEGF levels in the co-treatment groups at 48 h. However, this change was not statistically significant. No changes were observed in the VEGFA levels in response to ICG001, in the LPS-treated group, at the 24 h treatment. Additionally, we investigated whether stabilizing AJ-associated β-catenin can promote barrier phenotype. The barrier phenotype is defined by the loss of plasmalemma vesicle-associated protein (PLVAP), which mediates transcytosis. Retinal mRNA analysis for PLVAP showed an increasing trend with LPS treatment, which was significantly downregulated with ICG001 treatment after 48 h (Figure 8). Our results indicate that LPS-induced PLVAP expression is reduced with ICG001 treatment.

## 4. Discussion

The integrity of the inner BRB predominantly relies on the coordinated actions of the retinal neurovascular unit (NVU) [43]. Inflammation, hypoxia, and hyperglycemia can damage the retinal NVU, compromising barrier function [44]. Apart from the vascular changes, pathological glial activation [45] and VEGF produced by Müller cells are significant contributing factors to retinal vascular leakage [46]. The molecular mechanisms of endothelial and epithelial cell AJ and TJ modulations have been extensively characterized on their contribution to paracellular permeability [47,48,49,50] and BRB disruption [51], which is a major hallmark of retinal diseases like uveitis, DR, retinopathy of prematurity, and age-related macular degeneration [52]. In the current study, we centered our attention on the potential benefits of pharmacologically targeting nuclear β-catenin by ICG001 in stabilizing endothelial AJs and inhibiting Wnt signaling, consequently fortifying TJs to prevent LPS-induced retinal vascular permeability and inflammation.

In our study, we noted that the HREC barrier disruption induced by LPS affects β-catenin localization in AJ complexes, where it typically resides. Based on the literature, β-catenin thus translocated to the nucleus functions as a transcription factor [53]. We and others have demonstrated that nuclear β-catenin, in association with FoxO1/3a, suppress Cldn5 expression in ECs, which results in the loss of EC barrier function and increased vascular permeability [19,33,35]. Thus, β-catenin plays a major role in vascular homeostasis [54]. Previous studies have reported that interaction between β-catenin and VE-cadherin in ECs switches the permeable vasculature phenotype to a barrier state [55]. We previously reported that treatment with LPS does not lead to alterations in VE-Cadherin expression in HRECs, but it does diminish the localization of β-catenin within the AJs and decreases the expression of Cldn5 [25]. The literature indicates that LPS stimulation leads to GSK3β activation, in turn, phosphorylating and disrupting β-catenin from the AJs [56] and the Wnt signaling complex [26]. Nuclear β-Catenin is a transcriptional activator or a repressor of the TCF/LEF-1 family of DNA binding proteins, leading to the transcriptional modulation of several target genes, including c-myc, CCND1, and VEGF [57] as well TJ proteins, mainly Cldn5 [19,33,35,58]. While this is essential during retinal vascular development, abnormal activation of these genes during retinal barrier maintenance can lead to pathology [57].

VE-Cadherin clustering stimulates the expression of the TJ adhesive protein Cldn5 via the PI3K/Akt/FoxO/β-catenin pathway [19]. When VE-Cadhein inter-cellular contacts are disrupted, β-catenin enters the nucleus, resulting in transcriptional suppression of TJ claudins, particularly Cldn5 [19,35]. When AJs are intact, this repression is released, and Cldn5 expression in the TJs is maintained presumably by recruiting most β-catenin at the membrane and preventing its entry into the nucleus. Apart from its role in AJs, β-catenin is also an integral component of the Wnt signaling complex regulating transcription. It is well known that Wnt mediates β-Catenin nuclear signaling. Studies show that dysregulated Wnt activation and, thereby, nuclear β-Catenin-mediated transcription is associated with vascular eye diseases [59,60] and retinal inflammation [61], which may be partially related to EC dysfunction—mostly due to direct activation of β-Catenin of the Wnt signaling pathway in ECs and inflammatory cells and secretion of inflammatory cytokines and VEGF, etc. [26]. 

ICG001, a small molecule Wnt signaling modulator, elicits its effects by selectively binding to CREB-binding protein (CBP) and, thereby, disrupts its interaction with β-catenin and inhibits CBP function as a co-activator of Wnt/β-catenin-mediated transcription [62]. Consequently, ICG001 has the potential to relocate free unbound nuclear β-catenin back to the cytosol and, subsequently, to AJs. This phenomenon is supported by our immunostaining of β-catenin in HREC monolayers, where the distribution of β-catenin at junctions improved notably following treatment with ICG001, even in the presence of LPS insult. The reduction in albumin extravasation observed with ICG001 treatment may be associated with the enhanced adhesive function of β-catenin. Apart from this, ICG001-mediated removal of β-catenin-mediated transcriptional repression of claudins may have played a role in safeguarding the vascular barrier. 

Although we anticipated ICG001 to strengthen BRB in the LPS-induced retina, its potential direct effect on inflammatory cells remains unclear. Our retinal mRNA analysis revealed that ICG001 could significantly inhibit the inflammatory surge induced by LPS as evidenced by a decrease in expression of pro-inflammatory cytokines at both 24 and 48 h endpoints. The level of TNFα and MCP-1 was several folds higher at 24 h post LPS, and the fold change reduced at a 48 h time-point suggesting a reduction in the inflammatory surge at 48 h post LPS. These results indicate that ICG001 can potentially suppress LPS-induced retinal inflammation, presumably by inhibiting Wnt-associated nuclear β-catenin. Whether the effect of ICG001 on inflammation is direct or indirect through its effect on normalizing retinal ECs or through its effect on Wnt signaling is not revealed in our current investigation; hence there is a study limitation.

In a healthy eye, inner BRB is governed by regulated interplay between paracellular and transcellular mechanisms. While paracellular transport is primarily controlled by AJs and TJs, transcellular transport is influenced by PLVAP. Increased PLVAP expression is reported in human retinal capillaries correlating to microvascular leakage associated with DR [63]. Likewise, PLVAP absence is essential for the formation of mature barrier endothelium of the BRB [64]. Evidence suggests that VEGF’s major permeability-contributing mechanism of caveolae formation is mediated by PLVAP through possible reorganization in the cytoskeletal framework [65]. Owing to the dynamic nature of the cytoskeleton, it is quite possible that altered endothelial transcellular nature in pathology can, in turn, affect paracellular junctional protein expression. These interdependent mechanisms when altered can contribute to pathological permeability. Intriguingly, the level of VEGF-A declined initially, lower than the control group, at 24 h but increased several folds at the 48 h time point with LPS, implying that the vascular permeability we observed at the 24 h time point could be VEGF-independent. At the same time, we saw an increased expression of PLVAP 48 h after treatment, which is a major transcytosis mediator in endothelial cells and a structural component of fenestrae [66]. Studies have shown that VEGF upregulates PLVAP expression [67]. Interestingly, ICG001 treatment significantly reduced the expression of VEGF-A and PLVAP at the 48 h time point. It is unclear in our study how the initial decline in VEGF at 24 h and the upregulation at 48 h time points could correlate to the increase in PLVAP levels we have observed at 24 and 48 h. Also, how LPS can modulate PLVAP expression is another new line of investigation. It is plausible that the expression of PLVAP is transcriptionally regulated by nuclear β-catenin associated with the Wnt pathway, and its inhibition from ICG001 leads to a decrease in its expression. In support of this notion, an interesting study published by Wang et.al suggested that normalizing β-catenin signaling could potentially contribute to the partial conversion of permeable ECs to a barrier-type state as evidenced by a decline in PLVAP and an increase in Cldn5 levels in circumventricular organ and ocular vasculatures [55]. Alternately, increased β-catenin localization in the AJs in HRECs with ICG001 treatment is evidence of its increased association with VE-Cadherins. The limitation of the study is that it does not explain how inhibition of nuclear β-catenin increases its presence in the AJs. Nevertheless, our results show a potential mechanistic explanation, at least in part, of how ICG001 might protect BRB in a pathological setup.

**Figure 9:**
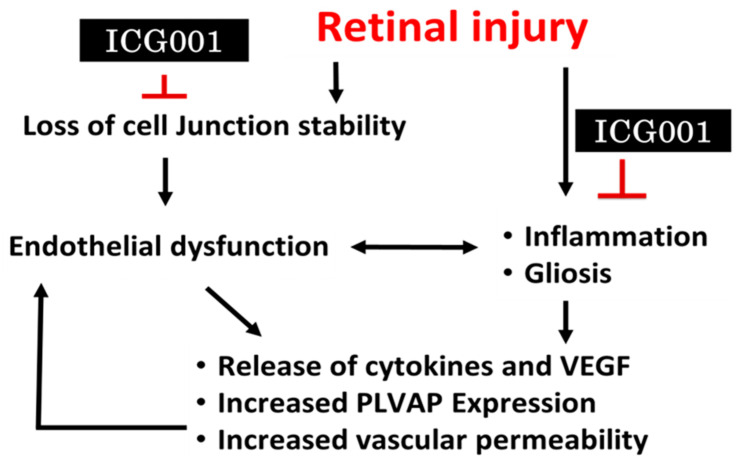
A schematic of the working hypothesis on the effect of β-catenin pharmacological modulation in retinal vascular permeability.

We observed acute reactive gliosis and microglia/macrophage activation in the mouse retinas with LPS stimulation, all of which were reversed with ICG001 treatment. While the initial inflammatory response promotes tissue repair, long-term microglial pro-inflammatory response can contribute to severe alterations in retinal vascular integrity and neurodegeneration [68]. ICG001 treatment can potentially inhibit microglial activation induced by LPS which is further supported by reduced inflammatory cytokine activation with ICG001 treatment. The effects of ICG001 need to be warranted by testing the drug in several other disease models including DR and other ocular permeability disorders as LPS-induced vascular permeability or inflammation in our model is acute and short-lived. Other ocular disease models of DR, such as streptozotocin-induced diabetes or spontaneously diabetic animal models could offer us a better understanding of how ICG001 could be beneficial in mitigating vascular permeability due to chronic inflammation. Nevertheless, our study holds the strength of identifying a novel mechanism for inhibiting pathological vascular permeability and re-purposing a drug that has shown promising effects as a Wnt signaling inhibitor in several pre-clinical cancer models. Some of the limitations of the study follow: We chose a preventive approach rather than a treatment approach, thinking β-catenin changes occur acutely, right after the insult. Using the intravitreal treatment approach of ICG001 could have avoided any off-target effects. The possible mechanism of ICG001 in reducing vascular permeability is mentioned below (Figure 9). Likely, ICG001 may not inhibit VEGF or PLVAP directly, while the effects we observed in our study most likely could be indirect effects of ICG001 by mitigating inflammatory cell activation as a result of Wnt signaling inhibition, which are major producers of VEGF or cytokines that contribute to retinal vascular and neuronal damage. However, ICG001 indeed paves the way in understanding the role of β-catenin in vascular permeability and inflammation.

## 5. Conclusions

The ideal treatment for any retinal vascular disease would be a drug that inhibits a key pathogenic mechanism but does not impact physiological processes. ICG001 appears to hold promise in mitigating vascular leakage, although its use may entail some side effects. The use of ICG001 may greatly benefit compromised barrier integrity in ocular inflammatory diseases. Alteration of β-catenin distribution could serve as a novel cellular mechanism to tackle pathological retinal vascular permeability.

## Figures and Tables

**Table 1:** List of primary and secondary antibodies used in the study.

Antibody/Probe	Dilution	Company	Catalog No.	Experiment
Primary Antibodies				
β-Catenin	1:200	Cell Signaling, (Danvers, MA, USA)	9562	Immunostaining
Albumin	1:200	Bethyl Laboratories, (Montgomery, TX, USA)	A110-134	Immunostaining
Iba-1	1:500	Wako, (Neuss, Germany)	019-19741	Immunostaining
F4/80	1:100	Abcam, (Waltham MA USA)	ab6640	Immunostaining
GFAP	1:500	Agilent Technologies, (Santa Clara, CA, USA)	Z033429–2	Immunostaining
Isolectin, GS-IB4 Alexa Fluor 594	1:200	Thermo Fisher Scientific	I21413	Immunostaining
		Secondary Antibodies		
Donkey anti-Sheep IgG Alexa Fluor 488	1:500	Jaxson Immune Research, (West Grove, PA, USA)	713-545-147	Immunostaining
Donkey anti-Rabbit IgG Alexa Fluor 488	1:500	Invitrogen, (Waltham, MA, USA)	A21206	Immunostaining
Donkey anti-Rat IgG Alexa Fluor 488	1:500	Invitrogen	A21209	Immunostaining

**Table 2:** List of mouse primer sequences used in the study.

Gene Name	Forward Primer	Reverse Primer
TNFα	GGTCCCCAAAGGGATGAGAA	TGAGGGTCTGGGCCATAGAA
IL1β	CCAAGCAACGACAAAATACC	GTTGAAGACAAACCGTTTTTCC
MCP-1	GGCTCAGCCAGATGCAGTTAA	CCTACTCATTGGGATCATCTTGCT
IL6	AGACAAAGCCAGAGTCCTTCAG	TGCCGAGTAGATCTCAAAGTGA
VEGF-A	AGCTCATGGACGGGTGAGG	CCTGGGACCACTTGGCAT
PLVAP	CGTCAAGGCCAAGTCGCT	CAGCAGGGTTGACTACAGGG
GAPDH	TGGTGAAGGTCGGTGTGAAC	CCATGTAGTTGAGGTCAATGAAGG
HPRT	GAAAGACTTGCTCGAGATGTCATG	CACACAGAGGGCCACAATGT

## Data Availability

Data will be made available upon formal request.
